# Analysis of a mechanistic model of corals in association with multiple symbionts: within-host competition and recovery from bleaching

**DOI:** 10.1093/conphys/coac066

**Published:** 2022-10-11

**Authors:** Alexandra Lynne Brown, Ferdinand Pfab, Ethan C Baxter, A Raine Detmer, Holly V Moeller, Roger M Nisbet, Ross Cunning

**Affiliations:** Department of Ecology, Evolution, and Marine Biology, University of California, Santa Barbara, Santa Barbara, CA 93106, USA; Department of Ecology, Evolution, and Marine Biology, University of California, Santa Barbara, Santa Barbara, CA 93106, USA; Department of Ecology, Evolution, and Marine Biology, University of California, Santa Barbara, Santa Barbara, CA 93106, USA; Department of Ecology, Evolution, and Marine Biology, University of California, Santa Barbara, Santa Barbara, CA 93106, USA; Department of Ecology, Evolution, and Marine Biology, University of California, Santa Barbara, Santa Barbara, CA 93106, USA; Department of Ecology, Evolution, and Marine Biology, University of California, Santa Barbara, Santa Barbara, CA 93106, USA; Daniel P. Haerther Center for Conservation and Research, John G. Shedd Aquarium, Chicago, IL 60605, USA

## Abstract

Coral reefs are increasingly experiencing stressful conditions, such as high temperatures, that cause corals to undergo bleaching, a process where they lose their photosynthetic algal symbionts. Bleaching threatens both corals’ survival and the health of the reef ecosystems they create. One possible mechanism for corals to resist bleaching is through association with stress-tolerant symbionts, which are resistant to bleaching but may be worse partners in mild conditions. Some corals have been found to associate with multiple symbiont species simultaneously, which potentially gives them access to the benefits of both stress-sensitive and -tolerant symbionts. However, within-host competition between symbionts may lead to competitive exclusion of one partner, and the consequences of associating with multiple partners simultaneously are not well understood. We modify a mechanistic model of coral-algal symbiosis to investigate the effect of environmental conditions on within-host competitive dynamics between stress-sensitive and -tolerant symbionts and the effect of access to a tolerant symbiont on the dynamics of recovery from bleaching. We found that the addition of a tolerant symbiont can increase host survival and recovery from bleaching in high-light conditions. Competitive exclusion of the tolerant symbiont occurred slowly at intermediate light levels. Interestingly, there were some cases of post-bleaching competitive exclusion after the tolerant symbiont had helped the host recover.

## 1 Introduction

Coral reefs are of immense ecological and economic importance. They are the most diverse marine habitat per unit area (Knowlton *et al.*, 2010), and the ecosystem services they provide are estimated at $9.9 trillion in value (Costanza *et al.*, 2014). These ecosystem services include acting as a source of fish and invertebrates for food (Reaka-Kudla, 1997), as well as physically buffering wave action and reducing the risk of coastal flooding (Ferrario *et al.*, 2014). However, these reefs, and the biodiversity and ecosystem services they sustain, are now in decline due to threats to their foundation species.

The physical structure of coral reefs is created by scleractinian corals. These animals survive in the oligotrophic waters where reefs are found due to their photosynthetic symbionts (family Symbiodiniaceae) providing them with fixed carbon (Muscatine & Cernichiari, 1969). In stressful conditions (e.g. extreme temperatures, very high or low light), corals can lose their photosynthetic symbionts, a process known as coral bleaching. The increasing temperatures corals have experienced over the past several decades have lead to an increasing number of bleaching events, and these bleaching events are predicted to increase in severity and frequency (Hughes *et al.*, 2018). This is problematic because corals that have bleached suffer increased mortality, reduced growth and reduced fecundity, sometimes even after returning to an unbleached state (Baird & Marshall, 2002). Corals that bleach and do not recover their symbionts will eventually starve and die (Putnam *et al.*, 2017). The loss of live corals may slow or stop reef accretion (Putnam *et al.*, 2017), with significant downstream impacts on the biodiversity and ecosystem services dependent on the physical structure of coral reefs (Bellwood *et al.*, 2012, Hoegh-Guldberg *et al.*, 2007).

Coral bleaching depends on a variety of factors, including coral species and morphology (Baird & Marshall, 2002, McCowan *et al.*, 2002), habitat (Morikawa & Palumbi, 2019, Rowan *et al.*, 1997) and previous exposure to stress events (Safaie *et al.*, 2018). In particular, symbiont species has a strong effect on bleaching susceptibility (Cunning *et al.*, 2015, Morikawa & Palumbi, 2019, Rowan *et al.*, 1997, Silverstein *et al.*, 2017). For example, hosts with *Cladocopium* (formerly Clade C) symbionts bleached in response to cold and warm temperatures that hosts with *Durisdinium* (formerly Clade D) symbionts endured without bleaching (Silverstein *et al.*, 2017). There are several proposed mechanisms by which certain species of symbionts may be better able to tolerate stressful environmental conditions without bleaching. These ‘tolerant’ symbionts may resist bleaching simply by being better able to resist expulsion/death (Silverstein *et al.*, 2017), or they may modify their host’s physiology to be more stress-tolerant (Cunning & Baker, 2020). They may also be better able to function in the face of stress, for example by being better able to repair photodamage caused by light stress (Ragni *et al.*, 2010) or having a more stable photosynthetic apparatus (Tchernov *et al.*, 2004). Lower growth rates may also allow them to avoid resource-limitation that may lead to expulsion (Wooldridge, 2013). Their observed lower photosynthetic efficiency (in comparison to stress-sensitive symbionts) may also be related to their ability to tolerate thermal stress (Jones & Berkelmans, 2012).

Many coral species have been found to associate with multiple symbiont species, often at the same time (Silverstein *et al.*, 2012). When multiple symbiont taxa are present in a single coral colony, competitive dominance hierarchies (McIlroy *et al.*, 2019), environmental conditions (Silverstein *et al.*, 2017) and host selectivity (Blackstone & Golladay, 2018) can interact to shape the relative abundance of each symbiont. Thus, in nature, corals may be observed hosting mixtures of symbionts that range from mixtures of substantial proportions of multiple taxa (Fay & Weber, 2012, Rose *et al.*, 2021) to communities that are dominated by one symbiont type (Lee *et al.*, 2016, Mieog *et al.*, 2007). The adaptive bleaching hypothesis posits that by bleaching in response to stress, corals can form new associations with symbionts more suited to their new environmental conditions (Buddemeier & Fautin, 1993). Indeed, an increased proportion of thermally tolerant symbionts has been shown to increase coral bleaching resistance, while exposure to more extreme stress increased the proportion of these thermally tolerant symbionts in the host (Cunning *et al.*, 2015). This suggests that rare symbionts in corals could provide an advantage in the face of stressful conditions, where these symbionts might suddenly become highly beneficial partners (Ziegler *et al.*, 2018). However, other evidence suggests that rare symbionts, while common, are generally transient and have little effect on their coral host (Lee *et al.*, 2016).

Theoretical models provide an efficient avenue to investigate the consequences of corals associating with multiple symbiont species. Work by Ware *et al.* (1996) investigated the adaptive bleaching hypothesis using a Lotka-Volterra model of symbiont competition within hosts, assuming a trade-off between thermal tolerance and competitive ability. van Woesik *et al.* (2010) investigated within-host symbiont dynamics when symbionts have different light and temperature optima. By using Gaussian functions to define resource use, they were able to model symbionts with different degrees of generalism. Within-coral symbiont dynamics have also been modeled in the context of reef population dynamics and symbiont evolution by Baskett *et al.* (2009, 2010), using different temperature-dependent growth rates for different symbionts. So far, models of the within-host dynamics of multiple symbionts have generally required assumptions about the relationship between stress tolerance and competitive ability, in part because quantitative models explicitly incorporating the physiological rates that control within-coral host-symbiont interactions have only recently become available, notably though Dynamic Energy Budget (DEB) models incorporating syntrophic symbiosis.

Kooijman (2001) first proposed using concepts from DEB theory to model the coral holobiont, proposing the concept of ‘sharing the surplus’, whereby host and symbiont act selfishly, only transferring surplus metabolic products they cannot use themselves. Muller *et al.* (2009) developed the first full quantitative implementation of Kooijman’s concepts, with their approach subsequently used by Eynaud *et al.* (2011) to model coral bleaching. Cunning *et al.* (2017) developed a new simplified DEB model that shares many assumptions with the model of Eynaud *et al.*, but added an important new interaction, a positive feedback in which reduced carbon compounds rejected by the host fuel a carbon-concentrating mechanism that enhances CO_2_ supply to the symbiont. Adding this interaction has a profound impact on the dynamics, with the model showing that bleaching can be interpreted as a transition in the holobiont from a healthy (growing) state to an alternate steady state. Pfab *et al.* (2022) contains some detailed analysis of the dynamics in this model. These models focus on light as a potential stressor for corals, although corals also bleach due to high temperatures, as well as in response to other stressors, such as high levels of dissolved carbon dioxide, salinity extremes, certain environmental contaminants and cold stress (Baker & Cunning, 2015). We also chose to use light as our model of stress both for compatibility with prior DEB models and due to work that suggests that high light may be the proximate cause of bleaching in heat-stressed corals (Wooldridge, 2009). In general, we expect that heat stress should change the threshold light at which the events in the model happen, and may also impact other parameters such as increasing symbiont growth rate (Wooldridge, 2013). However, we expect the general conclusions of the model should be similar for heat and light stress.

Here, we extend the Cunning *et al.* DEB model to represent a coral in symbiosis with two symbiont species simultaneously. We model the within-host dynamics of stress-sensitive and stress-tolerant symbionts and the effect of these symbionts on host survival. We use the model to investigate the environmental conditions under which each symbiont is competitively dominant and the dynamics of their competitive interactions, as well as how the presence of both symbionts influences coral recovery from bleaching. These results shed light on the mechanisms of symbiont dynamics in corals, with implications for ecological responses to climate change. Our results may also be useful for conservation: to identify conditions under which corals with certain symbiont combinations are particularly at risk of bleaching, as well as provide information about how and when symbiont manipulation may function as a tool to improve resilience (National Academies of Sciences, Engineering, and Medicine, 2019).

## 2 Methods

### 2.1 The model

Our model is an extension of the DEB model of symbiosis between a single symbiont and a coral host created by Cunning *et al.* (2017). We describe the model fully in the Appendix. Briefly, the host and symbiont interact by ‘sharing the surplus’, where the host and symbiont provide the other with the excess nitrogen and carbon, respectively, remaining after they have used all they can for their own growth. As highlighted in the Introduction, there is also a positive feedback loop whereby hosts use excess carbon to power a carbon-concentrating mechanism that provides CO_2_ to symbionts. In many environments, the model exhibits bistability where healthy corals are characterized by nitrogen limitation of host and symbiont and bleached corals by carbon limitation of the host, and often also of the symbiont.

We have added the possibility of hosts having multiple symbionts at the same time. We assume these symbionts interact with each other indirectly, through the excess carbon each shares with the host, the surplus nitrogen each receives from the host and through absorbing some of the light available to the total symbiont population. The host is not able to directly favor one symbiont over the other, and instead allocates excess nitrogen and the CO_2_ gained by its carbon-concentrating mechanism to symbionts proportional to the biomass of each symbiont type. There is some evidence from corals and related cnidarians that hosts in reality may have some ability to influence their symbiont distribution as well (Poland & Coffroth, 2017, Rose *et al.*, 2021, Xiang *et al.*, 2020). We chose not to model host influence on symbiont distribution in order to better understand the symbiont dynamics in the absence of the host favoring particular partner(s). These symbiont dynamics should provide the ‘raw material’ (symbiont population size) on which host ‘selection’ of symbionts can operate.

We investigate corals in symbiosis with a stress-tolerant and a stress-sensitive symbiont. We model the tolerant symbiont as having a higher capacity to resist the production of reactive oxygen species by excess light ($k_{\text {ROS}, T} = 250$ mol photons C-mol S$_T^{-1}$ d$^{-1}$; see Table A2 for parameter definitions) than the sensitive symbiont ($k_{\text {ROS}, S} = 80$ mol photons C-mol S$_S^{-1}$ d$^{-1}$). We also model the tolerant symbiont as having a lower maximum specific photosynthetic rate ($j_{CPm, T} = 1.0$ mol C C-mol S$_T^{-1}$ d$^{-1}$) and lower maximum specific growth rate ($j_{SGm, T} = 0.15$ d$^{-1}$) compared to the sensitive symbiont ($j_{CPm, S} = 2.8$ mol C C-mol S$_S^{-1}$ d$^{-1}$ and $j_{SGm, S} = 0.25$ d$^{-1}$).

Simulations were performed in R version 3.6.0 (R Core Team, 2015) using an extension of the coRal package (Cunning *et al.*, 2017). We have added extensions to allow for multiple symbionts and the presence of dissolved organic carbon to the package; the other extension, to initialize simulations with different states of the fluxes, we make available as supplementary code. Code to run the simulations and analyze the results is available at https://github.com/brownal/twosyms. Parameters for the simulations are given in Table A2.

#### 2.1.1 Environmental variables

We investigated coral-symbiont dynamics across a range of light, nitrogen and prey levels. We modeled light levels ranging from 5 to 60 mol photons/m$^2$/d, to simulate both normal and stressful conditions. The light levels that a coral experiences vary with its depth, the season and various other factors, but a range of 5–35 mol photons/m$^2$/day probably captures a reasonable amount of that variation (Edmunds *et al.*, 2018, Lesser *et al.*, 2000). Lower light levels may be experienced, but we chose to set the lower bound of the range at 5 mol photons/m$^2$/day, to ensure that the model coral and symbionts would not die due to lack of light. The upper range of the light levels we investigate simulate stressful conditions. Light is the only possible stressor in the model. Because in natural conditions multiple factors, particular heat and light, combine to stress corals (Brown, 1997), we must simulate somewhat higher values of light to produce the same effects without other stressors.

We simulated dissolved inorganic nitrogen levels ranging from $10^{-8}$ to $10^{-5}$ mol/l. We chose this range to capture both the range of nitrogen levels observed on coral reefs and the range of experimental nitrogen levels, which can be higher. Ammonium levels measured in Hawai‘i upwind of a reef flat tended to be around $10^{-7}$ mol/l, and mean ammonium levels $\pm $ standard error all fell within the range $10^{-8}$ to $10^{-6}$ mol/l (Stimson, 1997). Experimental nitrogen addition might perhaps produce concentrations in the low $10^{-5}$ mol/l range (Stimson, 1997), which the upper bound of the nitrogen levels we simulated should capture.

We simulated prey levels ranging from 0 to $4 \cdot 10^{-7}$ C-mol/l. Zooplankton larger than 64 $\mu $m were found to reach concentrations up to around $2 \cdot 10^{-7}$ C-mol/l at the Great Barrier Reef, with concentrations as low as around $10^{-8}$ C-mol/l also observed (Roman *et al.*, 1990). We expanded this range somewhat in our investigations based on Cunning *et al.* (2007), who model high levels of prey at $4 \cdot 10^{-7}$ C-mol/l and prey as low as 0 C-mol/l.

### 2.2 Determining competitive dominance

We randomly drew 20,000 combinations of the environmental variables (light, dissolved inorganic nitrogen and prey) from a uniform distribution with the ranges discussed above. To determine which symbiont was competitively dominant for each combination of environmental conditions, we initialize the model with 0.5 C-mol each of sensitive and tolerant symbionts, and 1 C-mol of host biomass. All other fluxes and state variables were set at the default initializations in the coRal package, which correspond to an initially healthy, unbleached state.

If, after 100 simulated days, the host growth rate was negative or zero, we assumed the host was dead and classified the outcome of competition as ‘no survival’. If the host was alive and the symbiosis was functional at 100 days, we continued the simulation until we had reached 10 simulated years. Then we classified the symbiont that was numerically more abundant as the superior competitor. (We never observed any cases where the symbionts were equally common.)

We then used linear discriminant analysis (LDA) using the MASS package (Venables & Ripley, 2002) to determine how environmental conditions affected competitive dominance. LDA takes a set of continuous independent variables and a categorical dependent variable. It creates linear combinations of the independent variables that can be used to separate predictor-space into regions corresponding to each category in the dependent variable. In our case, the independent variables are the environmental variables (light, nitrogen and prey) and the dependent variable is the ‘winner’ of symbiont competition: the sensitive symbiont, the tolerant symbiont or neither due to a lack of survival.

### 2.3 Rate of competitive exclusion

To determine the rate of competitive exclusion, we used the results of previous simulations determining competitive dominance. For environmental parameters where the sensitive symbiont was competitively dominant, we initialized the simulations with 0.99 C-mol of the tolerant symbiont, 0.01 C-mol of the sensitive symbiont and 1 C-mol of the host, with all other state variables and fluxes initialized with the coRal defaults. If the host was able to survive (showed positive growth after 100 days), we ran the simulation until either the sensitive symbiont grew to 99% of the total symbiont population, or 10 simulated years had passed. We plotted the time at which the sensitive symbiont reached 99% of the total symbiont population, if this occurred during the simulation.

For environmental parameters where the tolerant symbiont was competitively dominant, we repeated the same procedure, except that we initialized the simulations with 0.99 C-mol of the sensitive symbiont and 0.01 of the tolerant symbiont, and stopped the simulations when the tolerant symbiont had reached 99% of the total symbiont population.

In some cases, the host could not survive when initialized with 99% of the inferior competitor. We did not include these cases in the time to competitive exclusion. We show host ability to survive with different ratios of sensitive:tolerant symbionts in the supplement (Supplementary Figs. S1, S2 and S3).

### 2.4 Effect of tolerant symbiont on recovery from bleaching

We simulated the potential recovery of bleached hosts at 150,000 combinations of the environmental variables, randomly drawn from a uniform distribution over the ranges given above. At each combination of environmental variables, we first simulated potential recovery from bleaching with sensitive symbionts alone. We initialized these simulations in a bleached state by starting with a very low symbiont biomass, $10^{-4}$ C-mol, compared to the host biomass, 1 C-mol. We also initialized the host biomass formation rate to 0 day$^{-1}$ and the symbiont photosynthetic rate to 0 mol C/(C-mol symbiont $\cdot $ day). The initial values of the other fluxes were calculated based on these. We chose to use these fluxes to define the rest of the initializations based on Pfab *et al.*’s (2022) method for reducing the state space of models with dynamics on both slow time scales (symbiont and host biomasses) and fast time scales (the fluxes; see Supplementary Fig. S4). Conceptually, a low host biomass formation and low symbiont photosynthetic rate correspond to a host and symbiont that are probably in distress, and Pfab *et al.*’s approach shows that these initializations tend to correspond to bleached state.

**Fig. 1:**
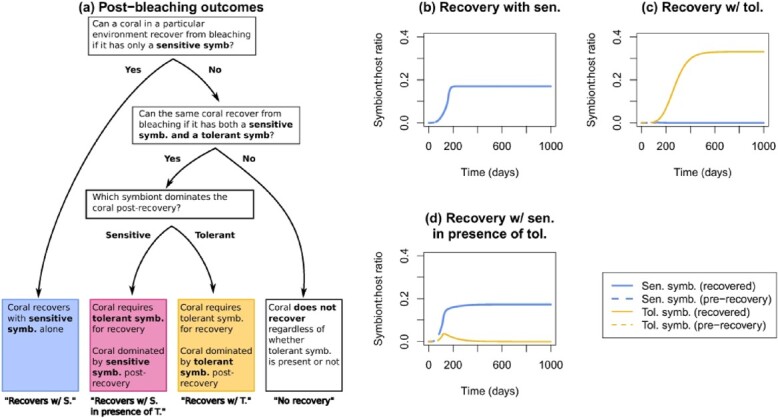
Classification of post-bleaching outcomes (a) and example simulations (b–d). Cases where hosts could recover from bleaching when in symbiosis with only sensitive symbionts were classified as ‘recovery with sensitive’ (b). Cases where the addition of a tolerant symbiont permitted host survival were classified as ‘recovers with tolerant’ if the tolerant symbiont was numerically dominant post-recovery (c) or ‘recovers with sensitive in the presence of tolerant’ if the sensitive symbiont outcompeted the tolerant post-recovery (d).

We ran the simulations with sensitive symbionts for 100 days, after which we checked if the host had returned to positive growth. If so, we marked the conditions as allowing recovery from bleaching with a sensitive symbiont alone. Otherwise, we tested whether the addition of a tolerant symbiont would allow recovery. We initialized the simulations as before, except that we started each symbiont with a biomass of $5 \cdot 10^{-5}$ C-mol, to give the same total biomass as before. At 100 days, we again checked for survival. If the host did not survive, we marked those environmental conditions as ‘no recovery’. Otherwise, we ran the simulations for an additional 900 days (total of 1000 days), to determine which symbiont was numerically dominant after recovery. We chose 1000 days to understand the medium to long-term effects of tolerant-assisted recovery, and in particular whether tolerant-assisted recovery could occur in cases where the tolerant symbiont was not competitively dominant post-recovery. Running the simulations for 10 years instead gave similar results (Supplementary Table S1). We compare the medium-term results with the short-term measures of the numerically dominant symbiont at the moment of recovery in the supplement (Supplementary Table S1 and Fig. S5).

To determine how the environment affected recovery outcomes, we then ran an LDA with the environmental variables as the independent variables. The dependent variable was the post-bleaching outcome: no recovery after bleaching (‘no recovery’), recovery possible with the sensitive symbiont (‘recovery with sensitive’) or recovery only possible when the tolerant symbiont was present. We further split the last category by which symbiont was numerically dominant after recovery: tolerant (‘recovery with tolerant’) or sensitive (‘recovery with sensitive in presence of tolerant’). See Fig. 1 for a summary of outcome classification and for example simulations for the three recovery outcomes.

## 3 Results

### 3.1 Within-host symbiont competition

**Fig. 2:**
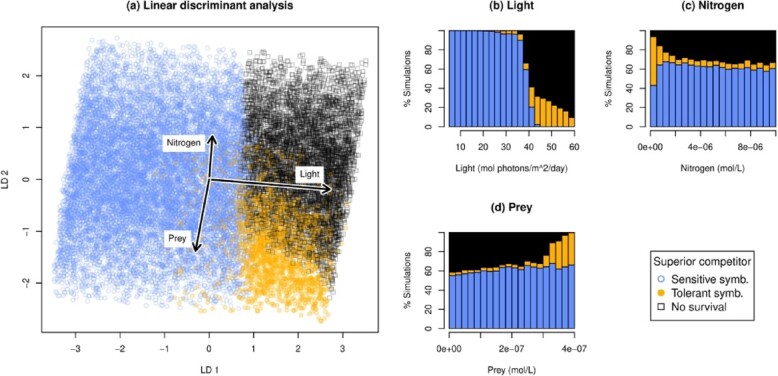
Effect of environmental conditions on within-host symbiont frequencies. (a) LDA of environmental conditions where sensitive or tolerant symbionts dominate the host. Sensitive symbiont dominance is marked with blue open circles and tolerant symbiont dominance by orange closed circles. Black open squares indicate conditions where the host cannot survive or maintain a functional symbiosis. Axes are the linear discriminants. LD1 explains 93.5% of the between-class variance; LD2 explains 6.5%. Arrows show the original environmental variables. (b–d) Distribution of symbiont dominance vs light (b), nitrogen (c) and prey (d). Blue bars at the bottom of the stacked histogram represent sensitive symbiont dominance; orange, middle bars tolerant symbiont dominance; and black, top bars no survival/dysfunctional symbiosis. Percentages are out of simulations in the environmental parameter range for each bar.

At low to moderate light, sensitive symbionts outcompete tolerant symbionts in hosts, while at high-light-tolerant symbionts outcompete sensitive (Fig. 2a and b). Prey and nitrogen have comparatively smaller effects on the outcome of competition (Fig. 2c and d), but become important under high light, where corals survive and maintain functional symbiosis with tolerant symbionts only when nitrogen is low and prey is high. We do not find any environmental conditions where both symbionts are equally good competitors; one always outcompetes the other.

### 3.2 Rate of competitive exclusion

**Fig. 3:**
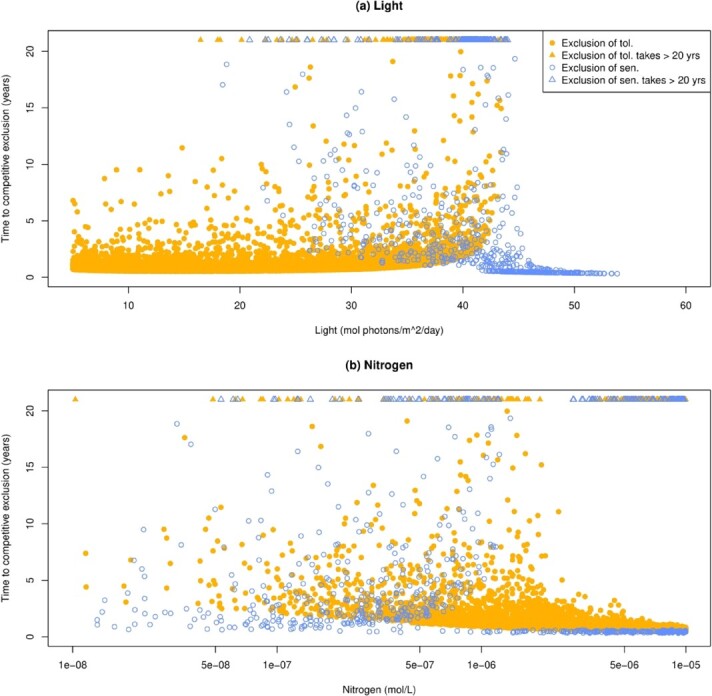
Effect of light and nitrogen on the time to competitive exclusion. Time is measured by the number of days/years it takes until the inferior competitor reaches 1% of the total symbiont population, when it began as 99% of the symbiont population. Triangles indicate cases where competitive exclusion was very slow, taking longer than 20 years. Cases shown are those where the host could survive and maintain a functional symbiosis when initialized with 99% of the inferior competitor. (a) Effect of light on time to competitive exclusion. (b) Effect of dissolved inorganic nitrogen on time to competitive exclusion. The x-axis here is on a log scale to better show what is happening at low nitrogen levels.

The rate of competitive exclusion (to 1% of the total symbiont population from 99% initially) is slow near 40 mol photons/m$^2$/d (Fig. 3a), around the light level where competitive dominance switches between sensitive and tolerant symbionts (Fig. 2b). Here, the inferior competitor can sometimes persist above 1% of the total symbiont population for over 20 years (assuming it started as 99% of the symbiont population initially). At lower or higher light levels, the rate of competitive exclusion is faster. The shortest time the sensitive symbiont persists above 1% of the total symbiont population is 115 days, while the shortest time for tolerant symbiont persistence is 193 days (Fig. 3).

Nitrogen availability has a similar effect on the time to competitive exclusion. The rate of competitive exclusion slows around $5 \cdot 10^{-7}$ mol/l, and is faster at lower or higher nitrogen levels (Fig. 3b). Prey availability has little effect on the rate of competitive exclusion (Supplementary Fig. S6).

### 3.3 Tolerant symbiont effects on bleached hosts

**Fig. 4:**
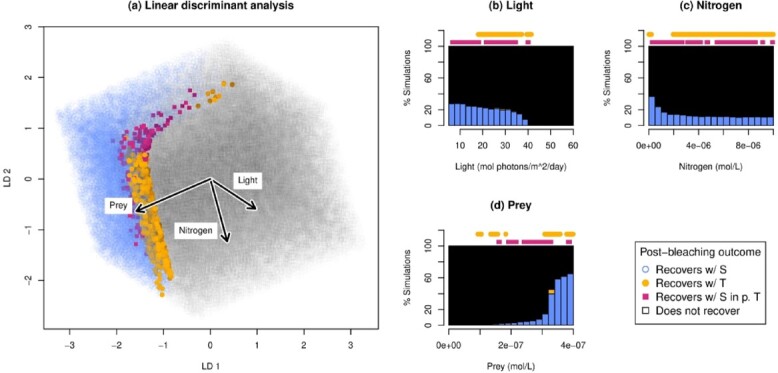
Distribution of post-bleaching outcomes. Recovery from bleaching is possible with the sensitive symbiont alone at low light and nitrogen levels and high levels of prey. The tolerant symbiont allows recovery along the ‘boundary’ between where recovery is possible with the sensitive symbiont alone and no recovery is possible at all, with higher light tending to favor recovery to a tolerant-dominant state (‘recovers with T’) vs a sensitive-dominated state (‘recovers with S in p. T’). (a) Points are projected onto the axes found by LDA. LD1 explains 99.7% of the between-class variance; LD2 explains 0.3%. (b–d) Distribution of post-bleaching outcomes across light levels (b), nitrogen levels (c) and prey levels (d). Blue bars at the bottom of the histograms indicate recovery with the sensitive symbiont alone. Orange bar in the middle (d only) indicates recovery with the addition of a tolerant symbiont to a tolerant-dominated state. Black bars indicate failure to recover. Dots along the top show rare outcomes that are not visible on the bar plot. Dots indicate tolerant-assisted recovery, leading to a sensitive-dominated state (pink squares, lower row) or a tolerant-dominated state (orange circles, upper row). Percentages are out of simulations in the environmental parameter range for each bar.

We find that bleached hosts fail to recover from bleaching to positive growth within 100 days in many environmental conditions that they can live in when healthy (Fig. 4). In particular, high prey levels are required for recovery in many cases. (We assume recovery that takes longer than 100 days indicates death in nature due to sustained biomass loss.) In the majority of cases where hosts can recover, the sensitive symbiont alone is enough to facilitate recovery. However, on the ‘boundary’ of survival, where light and nitrogen are slightly too high and food slightly too low to permit recovery with the sensitive symbiont alone, the addition of the tolerant symbiont may allow host recovery (Fig. 4). Cases of tolerant-permitted recovery that continue to be dominated by tolerant symbionts at 1000 days post-recovery tended to occur at higher light and nitrogen levels, while recovery to a sensitive-dominated state occurred at lower light and nitrogen levels. The direction of the effect of light on the numerical dominance of the tolerant symbiont is the same as for the competitive dominance of the tolerant symbiont (Fig. 2b), although the effect begins at lower light for recovery from bleaching. Interestingly, nitrogen acts in the opposite direction as it does for determining the competitive dominance of healthy hosts (Fig. 2c), although its effect on the outcome of recovery from bleaching is much less clear (Fig. 4c).

If hosts also have access to a source of dissolved organic carbon, the environmental conditions from which hosts can recover from bleaching is greatly expanded (Supplementary Fig. S7). The addition of a tolerant symbiont still permitted recovery in some conditions that hosts could not recover in with just the sensitive symbiont.

There is some debate about what exactly determines a ‘healthy’ coral in a model, and whether to exclude from this category corals that appear to be in a dysfunctional symbiosis. One potential indicator of a dysfunctional symbiosis is host carbon limitation (Wooldridge, 2013). In the supplement, we re-classify simulations with carbon limited hosts into the ‘no survival category’ (Supplementary Figs S8, S9 and S10). The results are similar, particularly with regard to the effect of light, although hosts in many more cases fail to recover from bleaching, making the environmental characteristics of tolerant-assisted recovery harder to determine. The effect of prey on competitive dynamics in healthy hosts is also diminished (Supplementary Fig. S8).

## 4 Discussion

Our model reveals mechanisms by which the availability of multiple mutualistic partners can allow their host to tolerate a wider range of environmental conditions, including acute stress events. This work fits into a broader body of research about multi-species mutualisms. It is well known that access to multiple mutualistic partners can expand an organism’s niche, if the partners are able to survive or to provide benefits in different environmental conditions (Giannini *et al.*, 2010, Oliver *et al.*, 2010). However, having access to both partners *simultaneously* is not always beneficial. Simultaneous partnership can be beneficial, for example, if each partner provides a unique benefit (Oliver *et al.*, 2010, Peay, 2016), if the presence of multiple partners alleviates saturation in host investment (Moeller & Neubert, 2016), or if the host is able to leverage the presence of an ‘outside option’ to induce more mutualistic behavior from a partner (Akçay & Simms, 2011). In contrast, partner diversity can be costly when mutualist competitive interactions exceed benefits (Van Nuland & Peay, 2020), when the less beneficial symbiont diverts resources from the more beneficial symbiont or when a partner that is a bad outside option weakens the host’s bargaining position in a resource exchange mutualism (Akçay & Simms, 2011).

In order to apply our theoretical understanding of multi-species mutualisms to conservation, we use a physiologically explicit model that accounts for the mechanism of mutualistic exchange. The coral host exchanges nitrogen for carbon produced by its symbionts, and stress can mean that tolerant symbionts are potentially better providers of carbon than sensitive symbionts. We find that access to a tolerant symbiont does indeed expand a coral’s niche, increasing its ability to recover from bleaching and survive high-light environments. However, both symbionts are never permanently maintained at appreciable frequencies in a constant environment (one always outcompetes the other). Functional symbiont coexistence is most likely at light levels, or stress levels more generally, close to the point where competitive dominance switches, particularly if frequent environmental variation around this point allows competitive dominance to alternate regularly. This was suggested by Safaie *et al.* (2018) as one possible mechanism for the increased coral survival seen in locations that experience frequent environmental variation.

Conversely, too much time in mild conditions may cause the loss of tolerant symbionts, or at least their decline to unhelpful levels when the next stress event occurs. Our results suggest that often months to years may be enough for a tolerant symbiont to decline past useful levels. Very extreme stress events followed by reversion to conditions that strongly favor sensitive symbionts might also cause the coral to temporarily continue to struggle (e.g. by experiencing lower growth rates; Jones & Berkelmans, 2010, Ortiz *et al.*, 2013), with their now large less beneficial tolerant symbiont population.

We also looked at how multiple symbionts influence host recovery from a stressful event (bleaching). Where recovery from bleaching is feasible, the sensitive symbiont is often sufficient for return to positive growth and a functional symbiosis. Occasionally, addition of the tolerant symbiont is required to allow survival. In some cases, once the tolerant symbiont has allowed the host to return to positive growth, it is outcompeted by the sensitive symbiont within 1000 days. These cases are fascinating from a mathematical perspective, where the combination of two symbionts produces an outcome that neither would have produced alone. They probably occasionally occur in reality, based on their occurrence along the ‘boundary’ in environmental space between recovery and failure to recover, even under conditions like the presence of dissolved organic carbon, which shift the boundary. They resemble successional dynamics, where a pioneer species paves the way for a later arriving species to flourish.

The dependence of coral survival on initialization in a bleached or unbleached state in certain environmental conditions makes analysis of the full behavior of the model challenging without a great deal of simulations. Pfab *et al.* (2022) have recently proposed a systematic method of exploring the outcomes of different initializations of DEB models, which may make future analysis of this and future DEB models of corals simpler.

Our model also has some limitations for dealing with the dynamics of real systems. First, the abstraction of all stress events as light stress may not be accurate for cases where the mechanism of stress is different. In particular, bleaching that occurs due to mechanisms other than photooxidative stress (for examples, see Baker & Cunning, 2015) is not well captured by our model. Second, we assume symbionts are always growing exponentially, meaning that as long as symbiont growth rates differ, competitive exclusion will eventually occur in a constant environment. As environmental variation is common in nature, our model can still reproduce coexistence in many natural circumstances. Third, we assume hosts and symbionts interact only via ‘sharing the surplus’ of nitrogen and carbon dioxide (hosts) and carbon (symbionts). In reality, host–symbiont interactions are more complex and may include host ‘control’ of the interaction via metabolic interactions (Xiang *et al.*, 2020), which may be evidenced by the observed symbiont specificity of certain host lineages (Poland & Coffroth, 2017, Rose *et al.*, 2021). Finally, we ignore all symbiont–symbiont interactions not caused by directly blocking light, so that we do not consider symbionts that are, e.g., slower growing but better able to divert resources from the host (all resources are allocated in proportion to symbiont population size). There may thus be more or fewer environmental regions that show either effective maintenance of both symbiont types or real coexistence.

By drawing on DEB theory, our model offers a mechanistic way to model the outcome of within-host competition and coral survival in multi-symbiont interactions. We see evidence that nearly stressful light environments may promote the maintenance of stress-tolerant and stress-sensitive symbionts, potentially improving corals’ chance of recovery from bleaching. Environmental variation that briefly moves corals into regimes where tolerant symbionts are competitively dominant may allow them to maintain a supply of these symbionts for future stress events. However, the slow rates of competitive exclusion under mild stress conditions (meaning tolerant symbiont populations may take a long time to grow) and the drop in host survival in high-stress conditions, suggest that experiencing occasional stress is not a guarantee that tolerant symbionts will be maintained or helpful in the future. While our model focuses on identifying qualitative patterns (Grimm *et al.*, 2005) in host–symbiont dynamics as a function of light stress, future empirical studies could validate our findings by seeking similar patterns in field and laboratory systems. For example, variable rates/outcomes of symbiont competitive exclusion (that depend upon environmental conditions) and/or symbiont dependence of coral host recovery from bleaching would all support our model’s predictions. Our findings have implications for identifying environments in which coral holobionts may maintain stress-tolerant phenotypes (as well as genotypes; Morikawa & Palumbi, 2019, Palumbi *et al.*, 2014) and for predicting tipping points in coral holobiont function that correspond to the loss of ecosystem function.

## 5 Appendix: DEB model

This model is an extension of Cunning *et al.* (2017) to include multiple symbionts. It uses DEB theory (Kooijman, 2009) to model the rates of biological process. Processes that convert various inputs into products are modeled as ‘synthesizing units’, e.g. the photosynthesis synthesizing unit is a function that takes as input the rates of light and CO_2_ arrival, and returns the rate of production of photosynthate.

The rate of product formation by a single-substrate synthesizing unit is modeled as $\left ( m^{-1} + x^{-1} \right )^{-1}$, where $m$ is the maximum rate of product formation and $x$ is the rate of substrate input (input flux). The two-substrate production flux is modeled as a parallel complementary synthesizing unit (Kooijman, 2009): $\left ( m^{-1} + x^{-1} + y^{-1} - (x + y)^{-1} \right )^{-1}$, where $m$ and $x$ are the same as the one-substrate equation and $y$ is the input flux for the second substrate.

### 5.1 State equations

Symbiont biomass, $S_i$ for symbiont $i$, is formed or lost according to the difference between the symbiont’s growth, $j_{SGi}$, and biomass loss (turnover), $j_{STi}$:

(A.1) \begin{align*}& \frac{dS_i}{S_i dt} = j_{SGi} - j_{STi}. \end{align*}

Similarly, the per-capita change in host biomass is given by the difference between host growth, $j_{HG}$, and host biomass turnover, $j_{HT}$:

(A.2) \begin{align*}& \frac{dH}{H dt} = j_{HG} - j_{HT}. \end{align*}

**Table A1:** Model fluxes, adapted from Cunning *et al.* (2017).

Symbol	Description	Units	Eq. no.
$j_X$	Prey assimilation (feeding) rate	C-mol X C-mol H$^{-1}$ d$^{-1}$	A.3
$j_N$	Nitrogen uptake rate	mol N C-mol H$^{-1}$ d$^{-1}$	A.4
$j_{HG}$	Host biomass formation rate	C-mol H C-mol H$^{-1}$ d$^{-1}$	A.5
$j_{HT}$	Host biomass turnover rate	C-mol H C-mol H$^{-1}$ d$^{-1}$	A.6
$r_{NH}$	Recycled nitrogen from host turnover	mol N C-mol H$^{-1}$ d$^{-1}$	A.7
$\rho _N$	Nitrogen shared with the symbiont	mol N C-mol H$^{-1}$ d$^{-1}$	A.8
$j_{eC}$	Excess carbon used to activate host CCMs	mol C C-mol H$^{-1}$ d$^{-1}$	A.9
$j_{CO_{2}}$	$CO_{2}$ input to photosynthesis	mol $CO_{2}$ C-mol H$^{-1}$ d$^{-1}$	A.10
$j_{Li}$	Light absorption rate for symbiont $i$	mol photons C-mol S$_i^{-1}$ d$^{-1}$	A.12
$r_{CH}$	Recycled $CO_{2}$ from host	mol $CO_{2}$ C-mol H$^{-1}$ d$^{-1}$	A.13
$r_{CSi}$	Recycled $CO_{2}$ from symbiont $i$	mol $CO_{2}$ C-mol S$_i^{-1}$ d$^{-1}$	A.14
$j_{CPi}$	Photosynthesis rate of symbiont $i$	mol C C-mol S$_i^{-1}$ d$^{-1}$	A.15
$j_{eLi}$	Light energy in excess of photochemistry for symbiont $i$	mol photons C-mol S$_i^{-1}$ d$^{-1}$	A.16
$j_{\text {NPQ}i}$	Total capacity of NPQ of symbiont $i$	mol photons C-mol S$_i^{-1}$ d$^{-1}$	A.17
$c_{\text {ROS}i}$	ROS production proportional to baseline for symbiont $i$	-	A.18
$r_{NSi}$	Recycled nitrogen from turnover of symbiont $i$	mol N C-mol S$_i^{-1}$ d$^{-1}$	A.19
$j_{SGi}$	Symbiont $i$ biomass formation rate	C-mol S$_i$ C-mol S$_i^{-1}$ d$^{-1}$	A.20
$\rho _{Ci}$	Fixed carbon symbiont $i$ shares with host	mol C C-mol S$_i^{-1}$ d$^{-1}$	A.21
$j_{STi}$	Symbiont $i$ biomass turnover rate	C-mol S$_i$ C-mol S$_i^{-1}$ d$^{-1}$	A.22

**Table A2:** Model parameters. Bold parameter values indicate different values for sensitive and tolerant symbionts. *Where two values are given, the first value is for the sensitive symbiont and the second for the tolerant.

Symbol	Description	Units	Value*
$n_{NH}$	N:C molar ratio in host biomass	-	0.18
$n_{NSi}$	N:C molar ratio in symbiont $i$ biomass	-	0.13, 0.13
$n_{NX}$	N:C molar ratio in prey biomass	-	0.2
$j_{HT}^0$	Maintenance rate of host biomass	C-mol H C-mol H$^{-1}$ d$^{-1}$	0.03
$j_{STi}^0$	Maintenance rate of symbiont $i$ biomass	C-mol S$_i$ C-mol S$_i^{-1}$ d$^{-1}$	0.03, 0.03
$\sigma _{NH}$	Proportion N turnover recycled in host	-	0.9
$\sigma _{CH}$	Proportion host metabolic $CO_{2}$ recycled to photsynthesis	-	0.1
$\sigma _{NSi}$	Proportion N turnover recycled in symbiont $i$	-	0.9, 0.9
$\sigma _{CSi}$	Proportion symbiont $i$ metabolic $CO_{2}$ recycled to photosynthesis	-	0.9, 0.9
$j_{Xm}$	Maximum prey assimilation rate from host feeding	C-mol X C-mol H$^{-1}$ d$^{-1}$	0.13
$K_X$	Half-saturation constant for prey assimilation	C-mol X L$^{-1}$	$10^{-6}$
$j_{Nm}$	Maximum host DIN uptake rate	mol N C-mol H$^{-1}$ d$^{-1}$	0.035
$K_N$	Half-saturation constant for host DIN uptake	mol N L$^{-1}$	$1.5 \cdot 10^{-6}$
$k_{CO_{2}}$	Efficacy of $CO_{2}$ delivery to photosynthesis by host CCMs	mol $CO_{2}$ mol C$^{-1}$	10
$j_{HGm}$	Maximum specific growth rate of host	C-mol H C-mol H$^{-1}$ d$^{-1}$	1
$y_{CLi}$	Quantum yield of photosynthesis for symbiont $i$	mol C mol photons$^{-1}$	0.1, 0.1
$y_C$	Yield of biomass formation from carbon	C-mol mol C$^{-1}$	0.8
$\bar {a}^*_i$	Effective light-absorbing cross-section of symbiont $i$	m$^2$ C-mol S$_i^{-1}$	1.34, 1.34
$k_{\text {NPQ}i}$	NPQ capacity of symbiont $i$	mol photons C-mol S$_i^{-1}$ d$^{-1}$	112, 112
$k_{\text {ROS}i}$	Excess photon energy that doubles ROS production in symbiont $i$, relative to baseline levels	mol photons C-mol S$_i^{-1}$ d$^{-1}$	**80, 250**
$j_{CPmi}$	Maximum specific photosynthesis rate of symbiont $i$	mol C C-mol S$_i^{-1}$ d$^{-1}$	**2.8, 1.0**
$j_{SGmi}$	Maximum specific growth rate of symbiont $i$	C-mol S$_i$ C-mol S$_i^{-1}$ d$^{-1}$	**0.25, 0.15**
$b_i$	Scaling parameter for bleaching response, symbiont $i$	-	5, 5

### 5.2 Coral animal fluxes

Corals assimilate prey from feeding at a rate $j_X$, which we assume increases with prey availability, $X$, following Michaelis-Menten kinetics, with a maximum rate $j_{Xm}$ and a half-saturation constant $K_X$.

(A.3) \begin{align*}& j_X = \frac{j_{Xm} \cdot X}{X + K_X} \end{align*}

Corals take up dissolved inorganic nitrogen, $N$, from the environment at a rate $j_N$, following Michaelis-Mentin kinetics, with a maximum rate $j_{Nm}$ and a half-saturation constant $K_N$.

(A.4) \begin{align*}& j_N = \frac{j_{Nm} \cdot N}{N + K_N} \end{align*}

Biomass formation requires carbon and nitrogen. It occurs at a maximum rate $j_{HGm}$. Carbon comes from the symbionts, $\rho _{Ci}$, and from prey, $j_X$, and can be converted to biomass with a yield of $y_C$. Nitrogen comes from the uptake of dissolved inorganic nitrogen, $j_N$, and prey, $n_{NX} j_X$, where $n_{NX}$ reflects the nitrogen:carbon ratio in prey, to convert from C-mol of prey to mol nitrogen. Hosts can also use nitrogen freed from their biomass via turnover, $r_{NH}$. Nitrogen is converted to biomass with a yield of $n_{NH}^{-1}$.

(A.5) \begin{align*} j_{HG} &=\! \left( \frac{1}{j_{HGm}} + \frac{1}{y_{C} \left( \sum{\left( \rho_{Ci} S_i/H \right)} + j_X \right)} +\right.\notag \\ &\qquad \frac{1}{\left( j_N + n_{NX} j_X + r_{NH} \right) n_{NH}^{-1}}\notag\\&\qquad\!\left. - \frac{1}{y_{C}\! \left( \sum\!{\left( \rho_{Ci} S_i/H \right)} \!+\! j_X \right) \!+\! \left( j_N \!\!+\! n_{NX} j_X \!+\! r_{NH} \right) n_{NH}^{-1}} \right)^{-1} \end{align*}

Host biomass turnover, $j_{HT}$ is assumed to occur at a constant rate $j_{HT}^0$.

(A.6) \begin{align*}& j_{HT} = j_{HT}^0 \end{align*}

Host biomass lost to turnover contains $j_{HT} \cdot n_{NH}$ mol nitrogen per C-mol host biomass, where $n_{NH}$ is the N:C molar ratio in host biomass. The host can recycle a fraction $\sigma _{NH}$ of this nitrogen for reuse.

(A.7) \begin{align*}& r_{NH} = \sigma_{NH} n_{NH} j_{HT} \end{align*}

We calculate the nitrogen rejection flux from the coral biomass synthesizing unit, $\rho _N$, as the excess nitrogen left after host biomass synthesis. Here $(x)_+$ represents the function $max(x, 0)$ and indicates that excess nitrogen can only be 0 or positive. Excess nitrogen is then given to the symbionts (Equation A.20).

(A.8) \begin{align*}& \rho_N = \left( j_N + n_{NX} j_X + r_{NH} - n_{NH} j_{HG} \right)_{+} \end{align*}

The carbon rejection flux from coral biomass SU, $j_{eC}$, is similarly calculated as the excess carbon left after host biomass synthesis.

(A.9) \begin{align*}& j_{eC} = j_X + \sum{\rho_{Ci} \frac{S_i}{H}} - j_{HG} y_{C}^{-1} \end{align*}

The excess carbon, $j_{eC}$ is then used to power the host carbon-concentrating mechanism (CCM), which delivers CO_2_ to symbiont photosynthesis at a rate of $j_{\text {CO\textsubscript {2}}}$ mol CO_2_ / (C-mol H $\cdot $ d). This CO_2_ is divided equally among symbionts by C-mol of symbiont biomass. Here $k_{\text {CO\textsubscript {2}}}$ represents the efficiency of the host CCM as the mol of CO_2_ it can concentrate with a mol of fixed carbon.

(A.10) \begin{align*}& j_{\text{CO\textsubscript{2}}} = k_{\text{CO\textsubscript{2}}} j_{eC} \end{align*}

### 5.3 Symbiont fluxes

The coral skeleton amplifies downwelling light, while symbiont cells will shade each other and reduce the light available. We calculate the light amplification factor, $A$, as in Cunning *et al.* (2017), modified to allow for multiple symbiont types. We assume all symbiont types contribute to shading in the same way, so that total symbiont biomass determines shading.

(A.11) \begin{align*}& A = 1.26 + 1.39 \cdot \exp{\left( -6.48 \cdot \frac{\sum{S_i}}{H} \right)} \end{align*}

Symbionts of type $i$ absorb light at a rate $j_{Li}$, which depends on their light-absorbing cross-section, $\bar {a}^*_i$, and the amplified downwelling light, $A \cdot L$.

(A.12) \begin{align*}& j_{Li} = A \cdot L \cdot \bar{a}^*_i \end{align*}

CO_2_ produced by host biological processes can be recycled to photosynthesis at a rate $r_{CH}$ mol CO_2_/(C-mol host $\cdot $ d). Hosts produce CO_2_ from biomass turnover at a rate of $j_{HT}$, as well as from host biomass formation at a rate $(1 - y_C) j_{HG} y_C^{-1}$. A fraction $\sigma _{CH}$ of this CO_2_ is then recycled to the symbiont.

(A.13) \begin{align*}& r_{CH} = \sigma_{CH} \left( j_{HT} + (1 - y_C) j_{HG} y_C^{-1} \right) \end{align*}

Similarly, a fraction $r_{CSi}$ of CO_2_ produced by the symbiont can be recycled to photosynthesis, with a rate of recycled CO_2_ of $r_{CSi}$. Carbon from baseline symbiont turnover, $j_{STi}^0$, and CO_2_ produced from symbiont growth may be recycled. Biomass lost due to ROS ($j_{STi} - j_{STi}^0$, see Equation A.22) is assumed to be expelled from the host and thus cannot be recycled.

(A.14) \begin{align*}& r_{CSi} = \sigma_{CSi} \left( j_{STi}^0 + (1 - y_C) j_{SGi} y_C^{-1} \right) \end{align*}

For photosynthesis, symbionts use the light absorbed, combined with CO_2_ input from the host carbon-concentrating mechanism (Eq A.10, above) as well as recycled CO_2_ produced by host and symbiont biomass turnover. The rate of photosynthesis, $j_{CPi}$, is modeled as a two-substrate (light and CO_2_) synthesizing unit with maximum photosynthetic rate $j_{CPmi}$. Maximum photosynthetic rate is thought to be lower in tolerant symbionts, so we model it as 2.8 mol C/(mol S $\cdot $ d) for sensitive symbionts and 1.0 mol C/(mol S $\cdot $ d) for tolerant symbionts.

(A.15) \begin{align*} j_{CPi} &= \left( \frac{1}{j_{CPmi}} + \frac{1}{y_{CLi} j_{Li}} + \frac{1}{(j_{\text{CO\textsubscript{2}}} + r_{CH}) \frac{H}{\sum{S_i}} + r_{CSi}} \right. \notag\\ &\qquad\left. - \frac{1}{y_{CLi} j_{Li} + (j_{\text{CO\textsubscript{2}}} + r_{CH}) \frac{H}{\sum{S_i}} + r_{CSi}} \right)^{-1} \cdot c_{\text{ROS}i}^{-1} \end{align*}

Light energy in excess of that used by photochemistry is produced at a rate $j_{eLi}$.

(A.16) \begin{align*}& j_{eLi} = j_{Li} - j_{CPi} y_{CLi}^{-1} \end{align*}

We model the rate of nonphotochemical quenching of the excess light, $j_{\text {NPQ}i}$, as a single-substrate synthesizing unit with maximum rate $k_{\text {NPQ}i}$.

(A.17) \begin{align*}& j_{\text{NPQ}i} = \left( k_{\text{NPQ}i}^{-1} + j_{eLi}^{-1} \right)^{-1} \end{align*}

Any excess light that is not quenched via nonphotochemical quenching leads to reactive oxygen species (ROS) production. We model this as $c_{\text {ROS}i}$, an increase in ROS production proportional to baseline levels of ROS production. $k_{\text {ROS}i}$ is the amount of unquenched light at which double the baseline ROS are produced. Tolerant symbionts have been suggested to be better at photorepair (Ragni *et al.*, 2010) or preventing the production of ROS (Tchernov *et al.*, 2004), so we model them as having a $k_{\text {ROS}}$ of 250 mol photons/(C-mol S $\cdot $ d) vs. sensitive symbionts’ $k_{\text {ROS}}$ of 80 mol photons/(C-mol S $\cdot $ d). Because ROS are produced locally, we model $c_{\text {ROS}i}$ and its effects on symbiont turnover (Equation A.22) as specific to each symbiont species, so tolerant and sensitive symbionts are not affected by the other’s ROS.

(A.18) \begin{align*}& c_{\text{ROS}i} = 1 + \frac{(j_{eLi} - j_{\text{NPQ}i})_{+}}{k_{\text{ROS}i}} \end{align*}

Nitrogen is recycled from symbiont biomass turnover at rate $r_{NSi}$. Only baseline biomass turnover, $j_{STi}^0$ produces nitrogen that can be recycled. A fraction $\sigma _{NSi}$ of the baseline turnover is actually recycled. To convert from C-mol of symbiont biomass in $j_{STi}^0$ to mol N, we use the symbiont biomass N:C molar ratio of $n_{NSi}$.

(A.19) \begin{align*}& r_{NSi} = \sigma_{NSi} n_{NSi} j_{STi}^0 \end{align*}

New symbiont biomass is formed via a two-substrate synthesizing unit, with maximum rate $j_{SGi}$. Sensitive symbionts generally have a higher maximum growth rate than tolerant, here we use a $j_{SGm}$ of 0.25 d$^{-1}$ for the sensitive symbiont and 0.1 d$^{-1}$ for the tolerant symbiont. The substrates of biomass formation are photosynthate and nitrogen. Symbionts can use nitrogen they recycle from their own biomass turnover ($r_{NSi}$) as well as that shared from the host ($\rho _N$). Nitrogen from the host is divided among symbiont species according to their biomass, so that each receives the same per C-mol amount of nitrogen.

(A.20) \begin{align*} j_{SGi} &= \left( \frac{1}{j_{SGmi}} + \frac{1}{y_C j_{CPi}} + \frac{1}{\left( \rho_N \frac{H}{\sum{S_i}} + r_{NSi} \right) n_{NS}^{-1}}\right.\notag\\ &\qquad\left. - \frac{1}{y_C j_{CPi} + \left( \rho_N \frac{H}{\sum{S_i}} + r_{NSi} \right) n_{NS}^{-1}} \right)^{-1} \end{align*}

Excess fixed carbon that the symbiont does not use for growth is shared with host at rate $\rho _{Ci}$.

(A.21) \begin{align*}& \rho_{Ci} = j_{CPi} - j_{SGi} y_C^{-1} \end{align*}

Symbiont biomass turnover occurs at a baseline rate of $j_{STi}^0$. Reactive oxygen species increase this turnover by $j_{STi}^0 b_i$ per unit of ROS present above baseline ($c_{\text {ROS}i} - 1$). $b_i$ here represents the scale of the bleaching response, so that symbionts with higher $b_i$ experience more biomass turnover relative to baseline as ROS increase.

(A.22) \begin{align*}& j_{STi} = j_{STi}^0 (1 + b_i (c_{\text{ROS}i} - 1)) \end{align*}

### 5.4 Carbon/nitrogen limitation

We can use the following formula to measure which substrate is limiting the rate of product formation for a two-substrate synthesizing unit, where $j_{S1}$ and $j_{S2}$ are the input fluxes for substrates 1 and 2, and $j_{Pm}$ is the maximum rate of product formation.

(A.23) \begin{align*}& \log \left( \frac{\min (j_{S1}, j_{Pm})}{\min (j_{S2}, j_{Pm})} \right) \end{align*}

Positive values of this expression indicate that substrate 2 is limiting, while negative values indicate that substrate 1 is limiting. A value of 0 means either that both substrates are equally limiting, or that the maximum rate of product formation has been reached.

## 6 Data Availability

Code can be found at https://github.com/brownal/twosyms

## Supplementary Material

Web_Material_coac066
