# Supplementary material for: Analysis of a mechanistic model of corals in association with multiple symbionts: within-host competition and recovery from bleaching
Source: Conserv Physiol. 2022 Oct 11;10(1):coac066. doi: 10.1093/conphys/coac066 (PMC9558299; doi:10.1093/conphys/coac066)
Supplement: Web_Material_coac066 [file web_material_coac066.zip › Supplement_coral_multiple_symbionts_DEB.pdf]

Supplement to "Analysis of a mechanistic model of corals in association with multiple symbionts"

AL Brown, F Pfab, EC Baxter, AR Detmer, HV Moeller, RM Nisbet, and R Cunning

Code

Code can be found at https://github.com/brownal/twosyms

Effect of sensitive:tolerant symbiont ratio on host survival

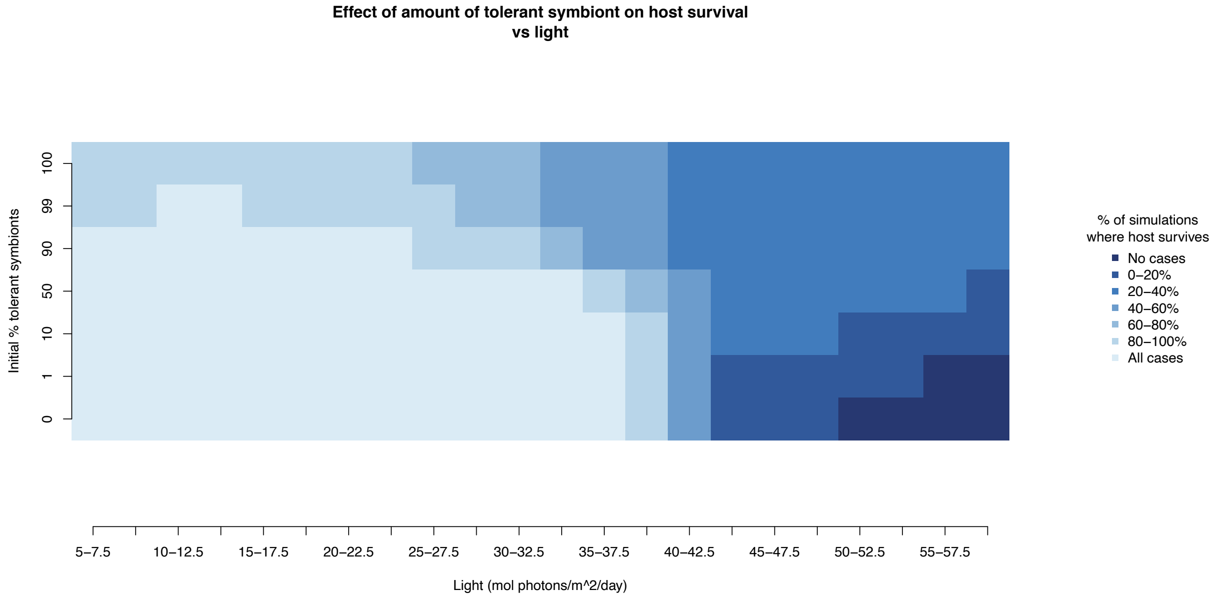

Figure S1: Effect of different amounts of tolerant symbiont on survival at different light levels. Host initialized in an unbleached state, with 1 C-mol total symbiont biomass and 1 C-mol total host biomass. Higher amounts of the tolerant symbiont increase host survival at high light, but decrease it elsewhere. At high light levels, often hosts with $\leq 10\%$ tolerant symbionts do not survive, where hosts with $\geq 50\%$ tolerant symbionts do.

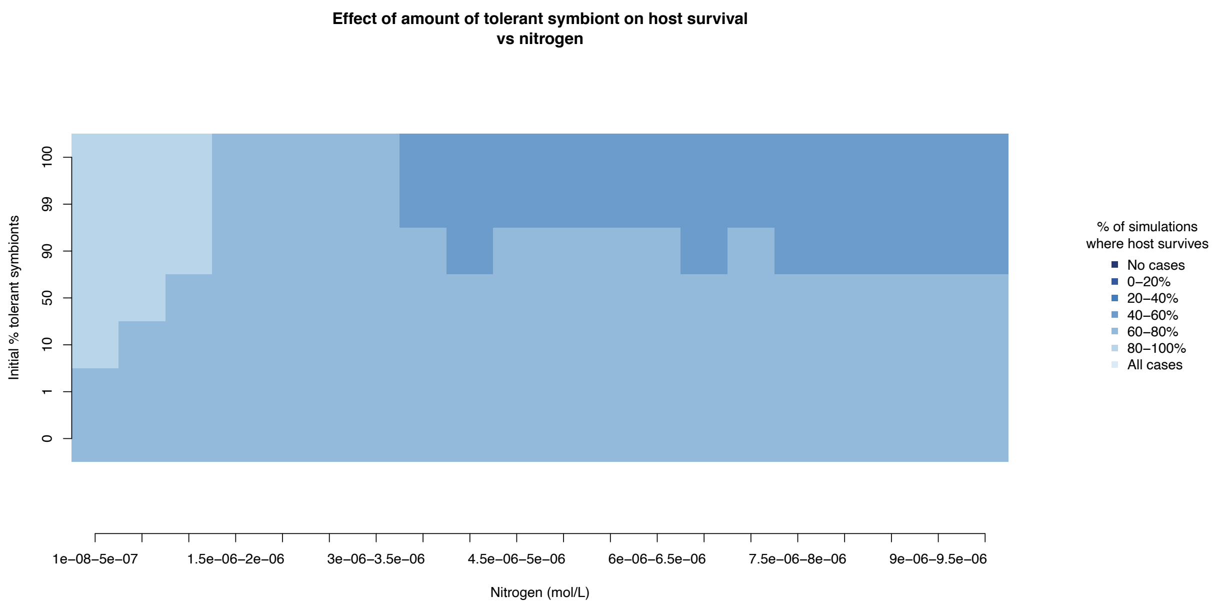

Figure S2: Effect of amount of tolerant symbiont on host survival across nitrogen levels. Hosts with more tolerant symbionts survive better at low nitrogen and worse at moderate to high nitrogen than hosts with more sensitive symbionts.

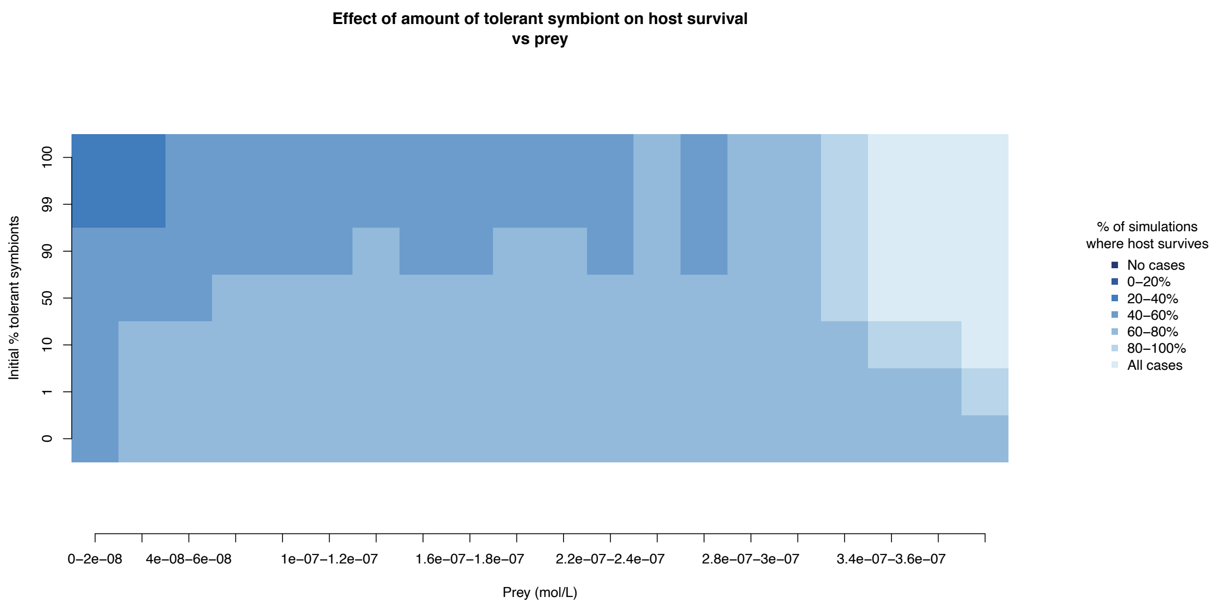

Figure S3: Effect of amount of tolerant symbiont on host survival at different levels of prey. Hosts with more tolerant symbionts survive better at high prey and worse at low to moderate prey than hosts with more sensitive symbionts.

9 Bleached flux initialization

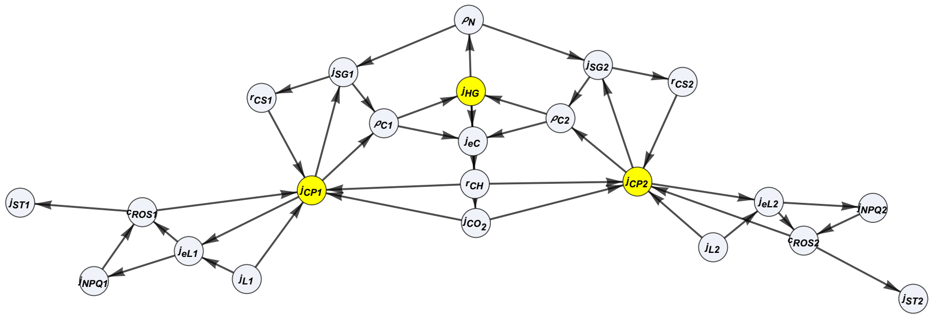

Figure S4: Network of fluxes in the model. Arrows point from inputs to synthesizing units (or other fluxes that depend on the inputs). The fluxes highlighted in yellow (j_{HG}, j_{CP1}, and j_{CP2}) can be used to calculate all the other fluxes in the model. Thus, these three fluxes can be used to initialize the model. The method for finding sufficient fluxes to define the rest of the model comes from Pfab et al. (in review). Code to identify these fluxes can be found in the supplementary code.

10 Tolerant-assisted recovery: symbiont competitive dynamics

More abundant symbiont			Percent of
At point of recovery	1000 days post-bleaching	10 years post-bleaching	simulations
Sensitive	Sensitive	Sensitive	19.7%
Sensitive	Tolerant	Tolerant	67.7%
Sensitive	Sensitive	Tolerant	4.1%
Sensitive	Tolerant	Sensitive	1.2%
Tolerant	Tolerant	Tolerant	6.1%
Tolerant	Sensitive	Sensitive	0%
Tolerant	Tolerant	Sensitive	1.2%
Tolerant	Sensitive	Tolerant	0%

Table S1: Changes in sensitive and tolerant symbiont relative abundance at different time points after tolerant-assisted recovery. Percentages given are out of total simulations where the tolerant symbiont was required for recovery from bleaching. Symbiont relative abundance is fairly consistent between 1000 days post-bleaching and 10 years post-bleaching (changes occur between 1000 days and 10 years in 6.5% cases). Symbiont relative abundance often changes between recovery and 1000 days post-bleaching (68.9% of cases), generally because the sensitive is more abundant at the point of recovery, but the tolerant symbiont outcompetes it afterwards.

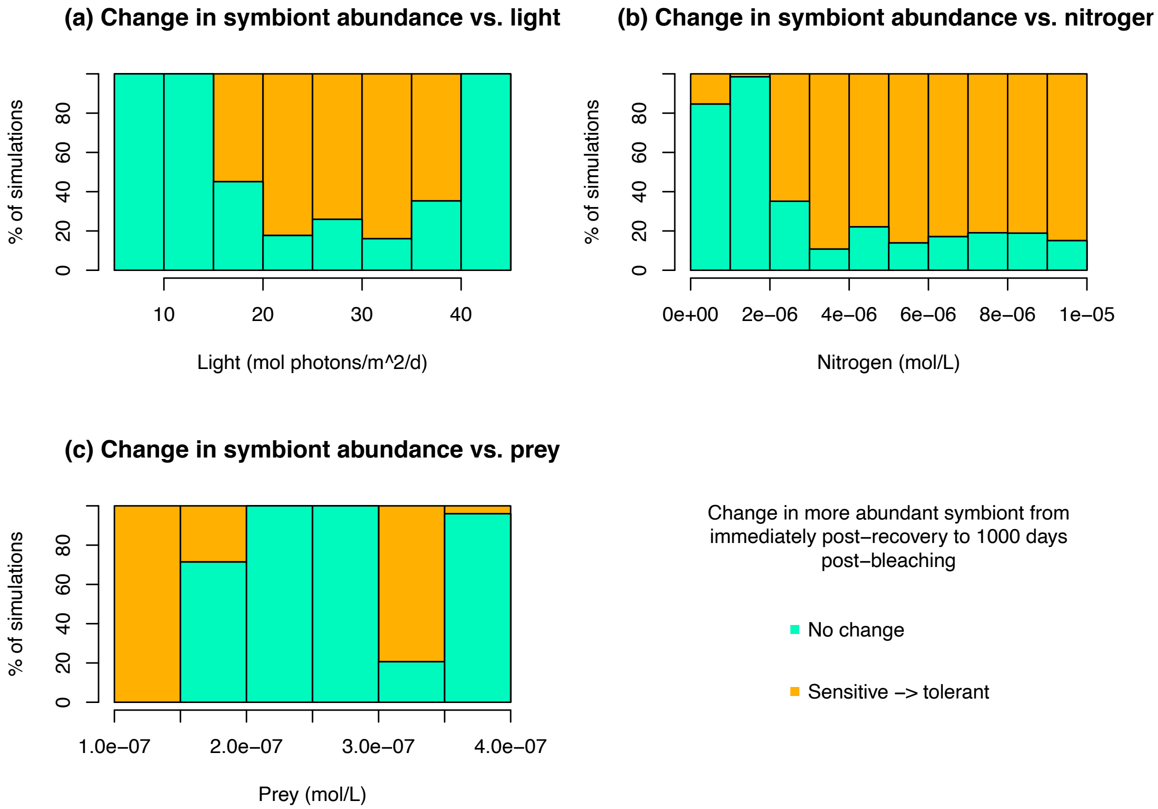

Figure S5: Change in symbiont relative abundance from immediately post-recovery to 1000 days post-bleaching. Percentages are out of simulations in the given environmental parameter range where the tolerant symbiont was required for recovery from bleaching. Green bars (lower bars) indicate simulations where the same symbiont was more abundant at both time points. Orange bars (upper bars and the entire leftmost bar for (c)) indicate simulations where the sensitive symbiont was more abundant at the point of recovery but the tolerant symbiont outcompeted it post-recovery. The reverse (where the sensitive symbiont outcompeted the originally more common tolerant) did not happen in the first 1000 days post-bleaching in any cases (see Table S1).

Competitive exclusion and prey

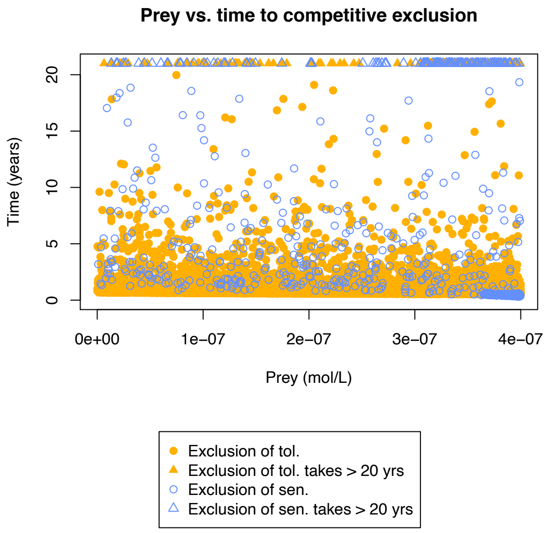

Figure S6: Prey availability has little effect on the rate of competitive exclusion.

Addition of dissolved organic carbon

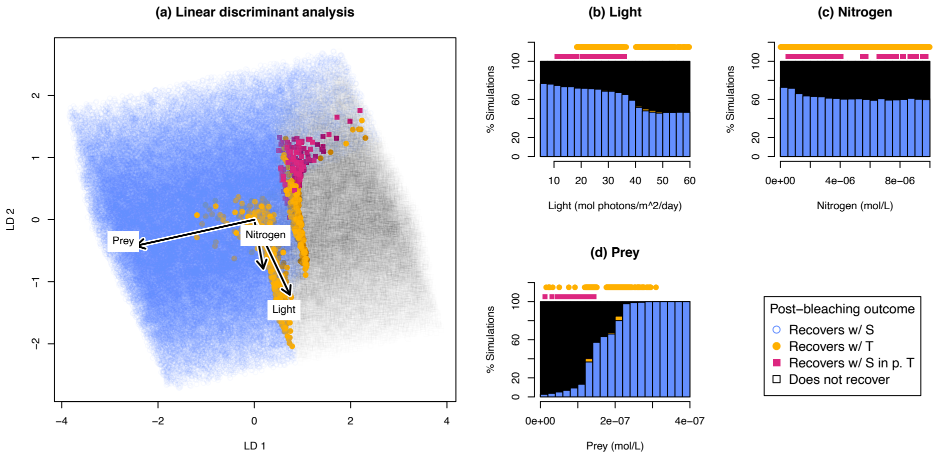

Figure S7: Recovery from bleaching when dissolved organic carbon is present. Host receives 0.0167 C-mol carbon/C-mol biomass, which goes directly into its biomass synthesizing unit (j_{HG}). The host is able to recover from bleaching in many more conditions than without dissolved organic carbon. (a) Linear discriminant analysis. LD1 explains $>$ 99.9% of the variance in post-bleaching outcome. (b-d) Effects of individual environmental variables on post-bleaching outcome. Cases where the tolerant symbiont enables recovery are marked on top of the histograms. (b) Light, (c) nitrogen, (d) prey. Percentages are out of simulations in the environmental parameter range for each bar.

Excluding symbioses where host is carbon limited

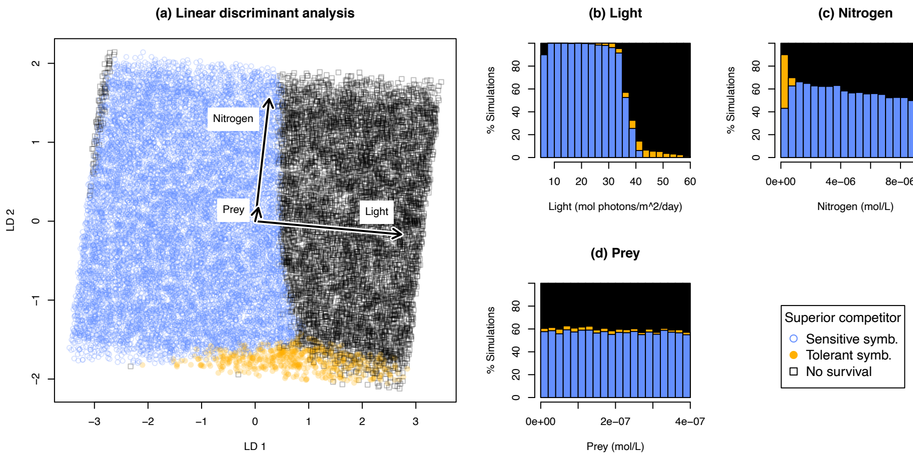

Figure S8: Effect of environmental conditions on within-host symbiont frequencies, when carbon limitation of the host is considered to indicate dysfunctional symbiosis and classified as "no survival." Qualitatively, the effects of light and nitrogen on survival and competition are similar to Fig 2. Compared with Fig 2, there are more cases of "no survival." In particular, many of the cases where the tolerant symbiont is competitively dominant are reclassified as "no survival," particularly at high prey levels. (a) Linear discriminant analysis of environmental conditions where sensitive or tolerant symbionts dominate the host. Sensitive symbiont dominance is marked with blue open circles, tolerant symbiont dominance by orange closed circles. Black open squares indicate conditions where the host cannot survive or maintain a functional symbiosis (i.e. the host carbon limited). Axes are the linear discriminants. LD1 explains 96.4% of the between-class variance; LD2 explains 3.6%. Arrows show the original environmental variables. (b), (c), and (d) Distribution of symbiont dominance vs light (b), nitrogen (c), and prey (d). Blue bars at the bottom of the stacked histogram represent sensitive symbiont dominance; orange, middle tolerant symbiont dominance; and black, top bars no survival/dysfunctional symbiosis. Percentages are out of simulations in the environmental parameter range for each bar.

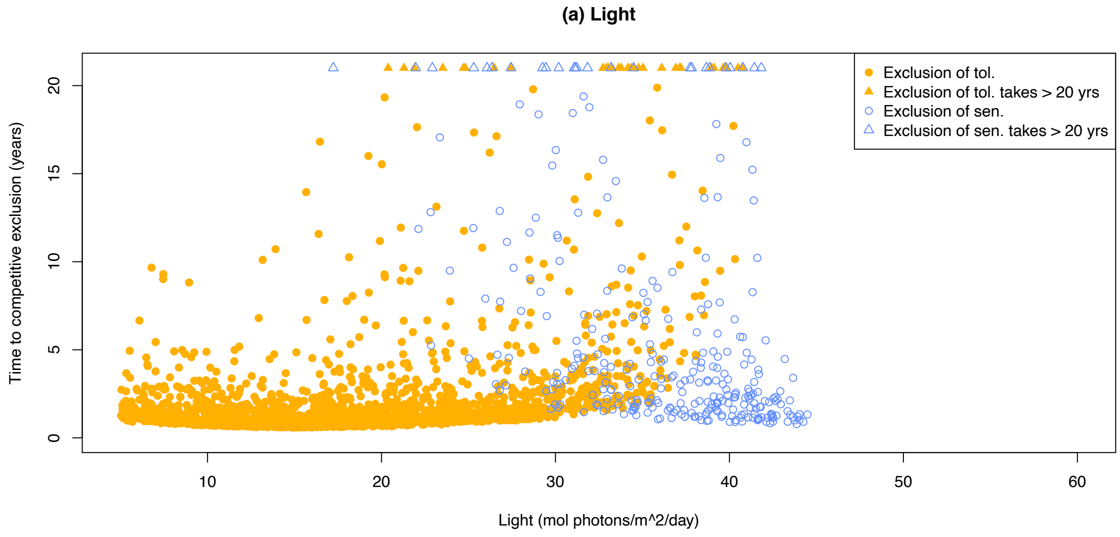

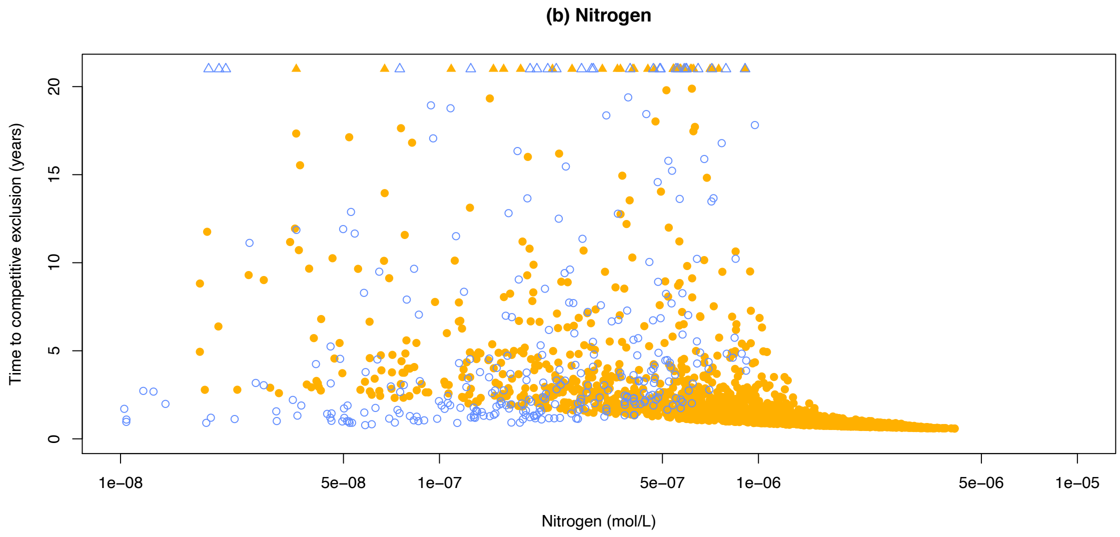

Figure S9: Effect of light and nitrogen on the time to competitive exclusion when carbon limitation of the host is considered to indicate dysfunctional symbiosis and classified as "no survival.". Compared with Fig 3, the effects of light and nitrogen are qualitatively similar, but the light level at which time to competitive exclusion is slowest (approximately 35 mol photons/m^2/day) is lower than in Fig 3 (approximately 40 mol photons/m^2/day). Cases shown are those where the host could survive and maintain a functional symbiosis (no host carbon limitation) when initialized with 99% of the inferior competitor. (a) Effect of light on time to competitive exclusion. (b) Effect of dissolved inorganic nitrogen on time to competitive exclusion. The x-axis here is on a log scale.

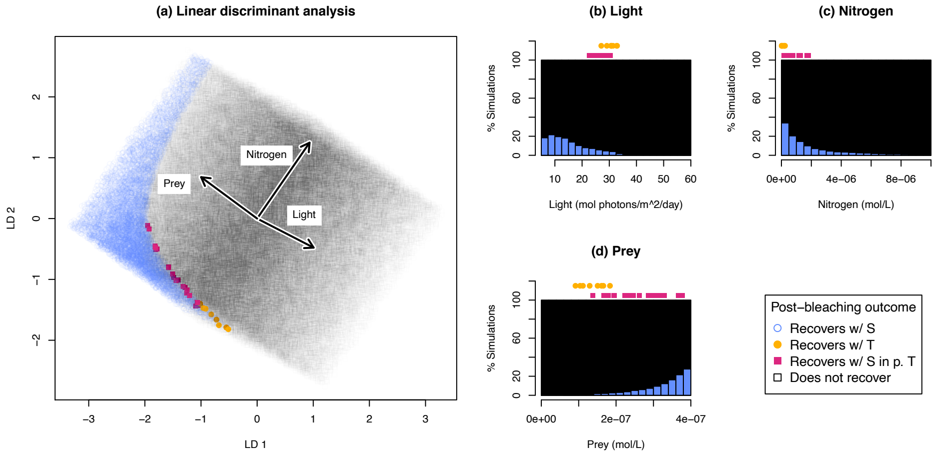

Figure S10: Distribution of post-bleaching outcomes when carbon limitation of the host is considered to indicate dysfunctional symbiosis and classified as "no recovery." Compared with Fig 4, there are far fewer cases of recovery, but the direction of effects of light, nitrogen, and prey on recovery remain qualitatively the same. (Low light and nitrogen increase recovery, as does high prey.) The addition of tolerant symbionts still allows some extra cases of recovery on the "boundary" of survival: at combinations of light, nitrogen, and prey that are just outside of what hosts with the sensitive symbiont alone cannot survive at. (a) Points are projected onto the first two axes found by discriminant analysis of principal components. LD1 explains 99.9% of the between-class variance; LD2 explains 0.1%. (b), (c), (d) Distribution of post-bleaching outcomes across light levels (b), nitrogen levels (c), and prey levels (d). Percentages are out of simulations in the environmental parameter range for each bar.
